# Evaluation of the sensitivity and specificity of a novel line immunoassay for the detection of criteria and non-criteria antiphospholipid antibodies in comparison to established ELISAs

**DOI:** 10.1371/journal.pone.0220033

**Published:** 2019-07-24

**Authors:** Markus A. Thaler, Andreas Bietenbeck, Udo Steigerwald, Thomas Büttner, Peter Schierack, Edelgard Lindhoff-Last, Dirk Roggenbuck, Peter B. Luppa

**Affiliations:** 1 Institut für Klinische Chemie und Pathobiochemie, Klinikum rechts der Isar der Technischen Universität München, München, Germany; 2 Zentrallabor, Zentrum Innere Medizin—A4, Universitätsklinikum Würzburg, Würzburg, Germany; 3 Medipan / GA Generic Assays GmbH, Dahlewitz, Germany; 4 Institut für Biotechnologie, Fakultät Umwelt und Naturwissenschaften, Brandenburgische Technische Universität Cottbus-Senftenberg, Senftenberg, Germany; 5 Coagulation Research Center CCB (Cardioangiologisches Centrum Bethanien), Frankfurt am Main, Germany; Universidad Nacional de la Plata, ARGENTINA

## Abstract

**Background:**

Persistent antiphospholipid antibodies (aPL) constitute the serological hallmark of the antiphospholipid syndrome (APS). Recently, various new assay technologies for the detection of aPL better suited to multiplex reaction environments than ELISAs emerged. We evaluated the diagnostic performance of such a novel line immunoassay (LIA) for the simultaneous detection of 10 different aPL.

**Methods:**

Fifty-three APS patients and 34 healthy controls were investigated for criteria (antibodies against cardiolipin [aCL], β2-glycoprotein I [aβ2-GPI]) and non-criteria aPL (antibodies against phosphatidic acid [aPA], phosphatidyl-choline [aPC], -ethanolamine [aPE], -glycerol [aPG], -inositol [aPI], -serine [aPS], annexin V [aAnnV], prothrombin [aPT]) IgG and IgM by LIA. Criteria aPL were additionally determined with the established Alegria (ALE), AcuStar (ACU), UniCap (UNI), and AESKULISA (AES) systems and non-criteria aPL with the AES system. Diagnostic performance was evaluated with a gold standard for criteria aPL derived from the results of the four established assays via latent class analysis and with the clinical diagnosis as gold standard for non-criteria aPL.

**Results:**

Assay performance of the LIA for criteria aPL was comparable to that of ALE, ACU, UNI, and AES. For non-criteria aPL, sensitivities of the LIA for aPA-, aPI-, aPS-IgG and aPA-IgM were significantly higher and for aPC-, aPE-, aAnnV-IgG and aPC- and aPE-IgM significantly lower than AES. Specificities did not differ significantly.

**Conclusions:**

The LIA constitutes a valuable diagnostic tool for aPL profiling. It offers increased sensitivity for the detection of aPL against anionic phospholipids. In contrast, ELISAs exhibit strengths for the sensitive detection of aPL against neutral phospholipids.

## 1. Introduction

Antiphospholipid syndrome (APS) is clinically characterized by recurrent venous or arterial thromboses or pregnancy complications [1]. To ensure comparability of clinical studies, within a research setting presence of APS is usually assumed when the criteria of the so-called Sydney-classification are fulfilled, i.e. persistent positivity for lupus anticoagulant (LA), anti-cardiolipin (aCL) or anti-β2-glycoprotein I (aβ2-GPI) IgG or IgM in addition to the typical clinical manifestations [2]. LA is determined in functional coagulation assays, whereas aCL and aβ2-GPI are commonly measured in ELISA systems [3].

Beyond the criteria anti-phospholipid antibodies (aPL) aCL and aβ2-GPI, aPL against several other anionic phospholipids (PLs) (e.g., phosphatidic acid, phosphatidyl-serine, -glycerol, -inositol), neutral PL (e.g., phosphatidyl-choline,–ethanolamine), and PL-binding proteins (e.g., annexin V, prothrombin) have been observed in APS [4, 5]. Their pathogenic role and clinical significance is not well understood and still a matter of debate [6–8]. These non-criteria aPL are also commonly detected with ELISA-based methods [5], which are generally less well evaluated than those for the criteria aPL [7, 9]. In order to obtain comprehensive aPL profiles for research and clinical purposes, new assay formats better suited to multiplexing than ELISA are badly needed. Consequently, various new assay technologies for detection of aPL have emerged. These include thin-layer chromatography immunostaining and line immunoassay (LIA) [10–12]. Employing a unique hydrophobic solid phase, the latter technique has already been successfully applied to analyze lipopolysaccharide- and glycolipid-(auto)antibodies [13]. In consideration of the similar physicochemical properties of the aforementioned antigens and PLs, LIA was identified as ideally suited for aPL detection and adapted to multiplexing.

Evaluation of diagnostic performances of novel technologies for aPL detection is challenging. Generally, methods for measurement of aPL still lack standardization [14–16]. Reference materials for aCL and aβ2-GPI have been suggested [17–21], but are controversially discussed and far from being fully accepted in daily practice. Moreover, candidate reference materials do not exist for non-criteria aPL. In addition, defining a proper gold standard for presence of APS in order to calculate sensitivities and specificities of aPL assays is complex as diseased or non-diseased status cannot be accurately determined without the results of the laboratory tests themselves [2].

The aim of the study was to investigate the diagnostic performance of a novel commercially available LIA enabling simultaneous detection of 10 different aPL. Results for the non-criteria aPL were evaluated with respect to the clinical diagnosis. Sensitivities and specificities for criteria-aPL were assessed using latent class analysis (LCA). We recently demonstrated [22], that LCA offers a statistically sound and innovative approach to explore the diagnostic performance of research biosensor assays for aPL detection [23, 24] despite the absence of proper reference material and method.

## 2. Materials and methods

### 2.1. Patients

Fifty-three sera of APS patients seen at the University Hospitals of Würzburg, Frankfurt, and the TU München were included in the study. All patients fulfilled the Sydney-criteria [2] as concluded from the local diagnostic work-up. Following immunoassays were used for initial evaluation of criteria aPL at the participating institutions: Varelisa ELISAs from Thermo Fisher Scientific (Freiburg, Germany) for 29, the ACL AcuStar (ACU) from Instrumentation Laboratory (Kirchheim, Germany) for 12, and the Alegria (ALE) system from Orgentec (Mainz, Germany) for 12 patients. The patient group consisted of 14 men and 39 women with a median age of 44 years. Primary APS had been diagnosed in 35 and secondary APS in 17 subjects. One patient could not definitely be assigned to either the primary or secondary group. 45 patients exhibited thromboembolic events (32 venous, 10 arterial, 3 venous and arterial), 3 suffered from pregnancy complications, and 5 subjects from a combination of both. Forty-six patients were positive for LA and according to the results of LCA (see below) 37, 12, 32, and 12 for aCL IgG and IgM, and aβ2-GPI IgG and IgM, respectively. Three patients exhibited double and 32 triple positivity as defined by Pengo et al. [25]. Two patients with missing data on LA status would either be double or triple positive. A healthy control group was recruited from the laboratory staff of the University Hospital of the TU München consisting of 9 men and 25 women (median age 41 years). Age and sex where not significantly different between patients and controls.

The investigated samples constitute a subgroup of the samples employed in a recent study [22], i.e. only samples with a sufficient amount of sample volume were included in the present work. As such, 53 of the 63 APS patients and all 34 healthy controls of [22] were investigated in the present study.

The study was approved by the local Ethics Committee (Ethikkommission der Fakultät für Medizin der Technischen Universität München, Prof. Dr. G. Schmidt, approval number 1558/06). All subjects gave written informed consent prior to inclusion. No financial compensation was provided.

### 2.2. aPL assays

Sera were analysed for aCL, anti-phosphatidic acid (aPA), -phosphatidyl-choline (aPC), -phosphatidyl-ethanolamine (aPE), -phosphatidyl-glycerol (aPG), -phosphatidyl-inositol (aPI), -phosphatidyl-serine (aPS), -annexin V (aAnnV), aβ2-GPI, and anti-prothrombin (aPT) IgG and IgM with the novel aPL LIA (GA Generic Assays (GA), Dahlewitz, Germany). The immobilized cofactors β2-GPI, prothrombin, and Annexin V are of human origin. PLs are immobilized on the polyvinylidene fluoride membrane of the LIA without any cofactor. Thus, the patient sample was the only possible source of β2-GPI for immobilized, negatively charged PLs. Processed strips were analyzed qualitatively, i.e. samples were classified as positive or negative by visually comparing (“eyeballing”) the obtained intensities of the respective bands to those of an interpretation template. Intensities of the reference bands on the interpretation template were established employing a scanner with the evaluation software Dr. DotLine Analyzer (GA). Optical density values equal to or above 50 score positive. This cut-off was determined by calculating the 99% percentile of 150 apparently healthy individuals as recommended by the international classification criteria for aPL testing [2, 26] and the Clinical and Laboratory Standards Institute (CLSI) guideline C28-A3. Using this cut-off, good agreement with qualitative data obtained by ELISA for the detection of aCL and aβ2-GPI was shown [27]. Linearity of dilution with optical density values was demonstrated in the range from 10 to 80 optical density units for most of the aPL-positive samples.

In addition, criteria aPL (aCL and aβ2-GPI IgG and IgM) were determined with four well-established immunoassay systems in all included sera: ALE, ACU, UniCAP 250 (UNI) from Thermo Fisher Scientific (Freiburg, Germany), and AESKULISA (AES) from Aesku.diagnostics (Wendelsheim, Germany). All commercial aβ2-GPI assays employed the human protein as antigen. Detection of aCL was performed with human β2-GPI in the ALE, ACU, and AES systems and bovine β2-GPI in the AES ELISA. IgG and IgM isotypes of the non-criteria aPL (aPA, aPC, aPE, aPG, aPI, aPS, aAnnV, aPT) were additionally measured with the AES system in all sera with sufficient sample material left after performing the analyses mentioned above. The wells of the AES assays for aPA, aPC, aPE, aPG, aPI, and aPS contained human β2-GPI as cofactor in addition to the respective PL. AES assays for aAnnV and aPT employed the human protein as antigen. Detailed information on the interpretation of the results obtained by the comparative immunoassay systems is given in Supporting Information S1 Table.

Samples were stored at -80°C in separate aliquots. For each measurement, a fresh aliquot was thawed. An explicit quality control for sample stability, however, was not performed. The presented results from the ALE, ACU, UNI, and AES for the criteria aPL are the same results also employed in [20] and hence constitute “old” measurements. Only the LIA results and the AES results for the non-criteria aPL are “new” measurements.

### 2.3. Statistics

Diagnostic performance of the LIA for criteria aPL was assessed by employing a LCA-derived “gold standard” calculated as described previously [22]. Briefly, in the context of this work, LCA assigns the observed test results to an unknown (“latent”) binary variable representing presence or absence of the respective antibody. For calculation of the LCA-derived gold standard, the results of the ALE, ACU, UNI, and AES where used, i.e. antibody positive or negative according to the cut-off recommended by the manufacturers. The underlying statistical model is expressed by a likelihood function. Provided at least three different tests have been performed on the respective antibody, maximizing this likelihood function enables estimation of its parameters [22]. As a result, sera were assigned to either the antibody-positive or -negative group in a way to yield the observed test outcomes with the highest probability. This assignment was then considered as “gold standard” to evaluate the diagnostic performance of the LIA for the criteria aPL.

LIA results for non-criteria aPL were compared to the AES ELISAs. Inter-rater agreement was assessed via Cohen’s κ. Agreements with Cohens’s κ of ≥ 0.81, 0.61–0.80, 0.41–0.60, 0.21–0.40, and ≤ 0.20 were considered as very good, good, moderate, fair, and poor, respectively [28]. For calculation of sensitivities and specificities, affiliation of the serum to either the patient or control group at inclusion was considered as the gold standard.

Sensitivities and specificities were compared in the McNemar test (significance level 0.05). “R” version 3.2.0 (R Foundation for Statistical Computing, Vienna, Austria) was employed for statistical evaluations. The statistical approach is illustrated in Supporting Information S1 Fig.

## 3. Results

### 3.1. Results of patients and controls in the LIA

**Fig 1:**
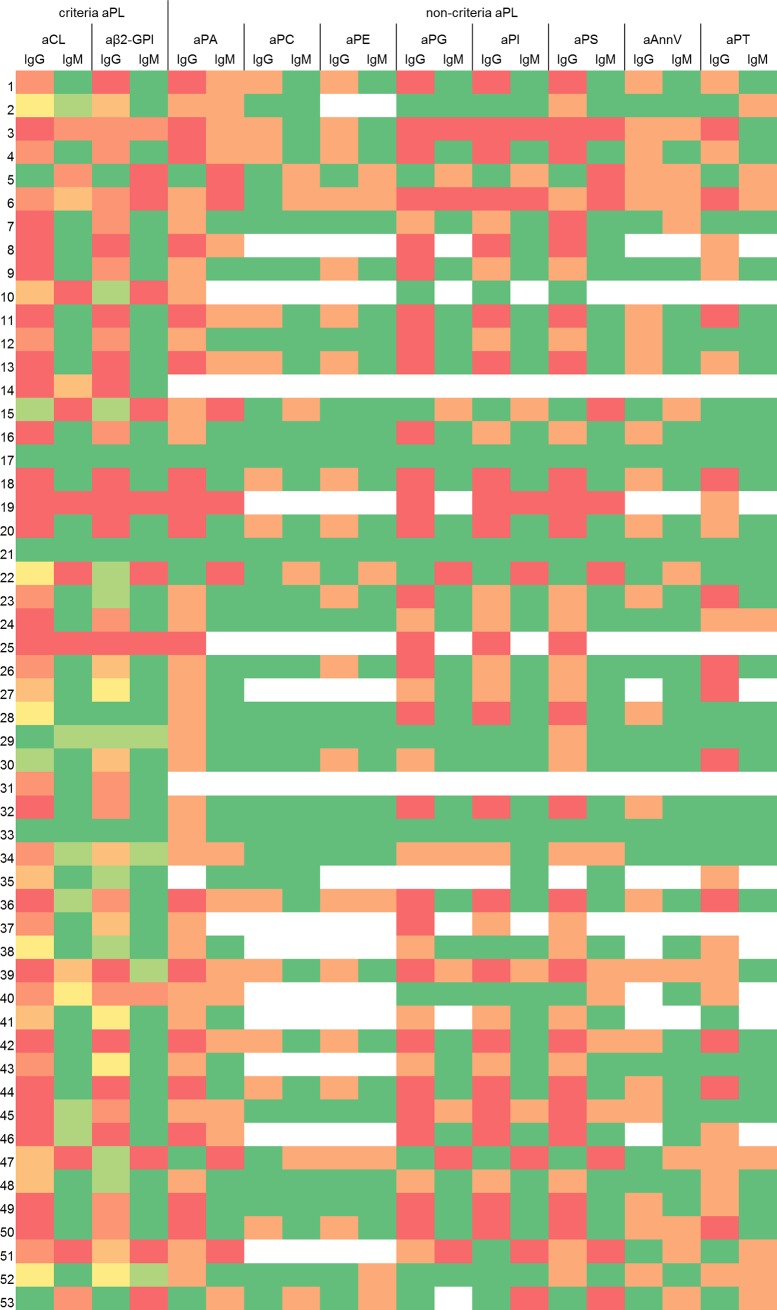
Heat map: Positivity of APS patients for criteria and non-criteria aPL in the various assays. Individual APS sera (n = 53) on the left. Investigated aPL on the top. Criteria aPL: dark green, no assay positive; bright green, one assay positive; yellow, two assays positive; bright orange, three assays positive; dark orange, four assays positive; red: all five assays positive (assays: ALE, ACU, UNI, AES, LIA). Non-criteria aPL: green, no assay positive; orange, one assay positive; red, both assays positive; white, not determined due to lack of sample material (assays: AES, LIA).

A comprehensive overview on the positivity of the APS patients for criteria and non-criteria aPL in the various assays is given in Fig 1. By LIA, 42 (79.2%) and 10 (18.9%) patients tested positive for aCL IgG and IgM, respectively, and 43 (81.1%) and 16 (30.2%) positive for aβ2-GPI IgG and IgM, respectively. With respect to non-criteria aPL, high positivity rates were found in the patient group for aPA IgG, aPS IgG, aPG IgG, and aPI IgG (n = 47 (88.7%), 43 (81.1%), 38 (71.2%), and 35 (66.0%), respectively), intermediate positivity rates for aPA IgM, aPT IgG, and aPS IgM (n = 25 (47.2%), 20 (37.7%), and 17 (32.1%), respectively), and low positivity rates for aPG and aPI IgM, aAnnV IgG and IgM, and aPT IgM (n = 11 (20.8%), 11 (20.8%), 4 (7.5%), 4 (7.5%), and 4 (7.5%), respectively). No patient exhibited a positive reactivity for aPC and aPE IgG or IgM.

**Fig 2:**
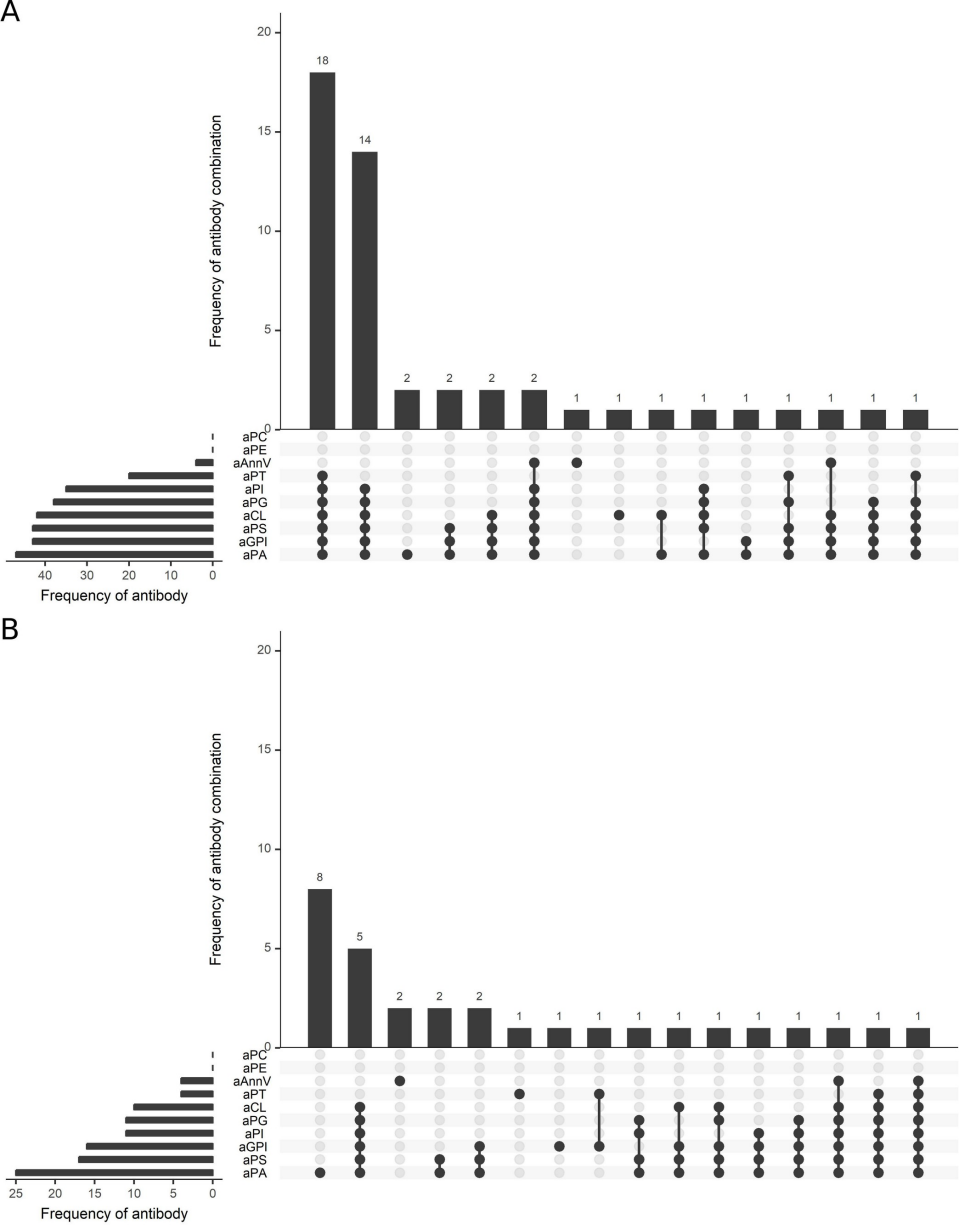
UpSet plot of the frequencies of aPL and aPL combinations in APS patients. Bars on the left depict the frequencies of the investigated aPL and bars above the frequencies of the various aPL combinations in the patient group (n = 53) for the (A) IgG and (B) IgM isotype. Filled circles indicate presence of the antibody in the respective group.

The aPL IgG combination found most frequently in the patient group was a concurrent positivity for criteria aPL (aCL and aβ2-GPI) and non-criteria aPL against anionic PLs (aPA, aPS, aPG, and aPI) with or without additional aPT (n = 18 (34.0%) and 14 (26.4%), respectively). Regarding IgM, the combination of positive criteria aPL and positive non-criteria aPL against anionic PL was observed frequently, too (n = 5 (9.4%)). The most frequent pattern for this isotype, however, was single positivity for aPA (n = 8 (15.1%)). All other observed aPL combinations were detected only in a minority of samples. An overview on the frequencies of the investigated aPL and their respective combinations is given in Fig 2.

Controls did not measure positive for aCL IgG and IgM and aβ2-GPI IgM. Three (8.8%) control sera were positive for aβ2-GPI IgG. When investigating the controls for non-criteria aPL, 4 (11.8%) positive reactions were found for aPT IgG, 3 (8.8%) for aAnnV IgM, 2 (5.9%) for aPA IgG, aPI IgG, and aPT IgM and 1 (2.9%) for aAnnV IgG and aPA IgM. Controls did not show positive staining for any of the other tested aPL.

### 3.2. Detection of low, medium, and high positive aPL sera in the LIA

Data on the detection rates for criteria aPL employing the LIA for sera with low, medium, and high positive titers in the comparative assays are given in Table 1. For aCL IgG and aβ2-GPI IgG and IgM 66.7–100.0% of the sera in the lowest terciles of the comparative assays were also found positive for the respective antibody in the LIA. Moreover, all sera located in the middle and highest terciles of the comparative assays for the aforementioned autoantibodies displayed positive reactions for the respective aPL in the LIA. With respect to aCL IgM, however, positivity rates in the LIA exhibited higher heterogeneity ranging from 16.7 to 100.0%, from 33.4 to 100.0%, and from 60.0 to 75.0% for sera in the first, second, and third terciles of the comparative assays, respectively.

**Table 1:** Detection of low, medium, and high positive sera for criteria aPL in the LIA.

aPL			ALE	ACU	UNI	AES
IgG	aCL	n	37	40	28	42
		T1	76.9%	71.4%	90.0%	78.6%
		T2	100.0%	100.0%	100.0%	100.0%
		T3	100.0%	100.0%	100.0%	100.0%
	aβ2-GPI	n	34	40	32	13
		T1	66.7%	85.7%	100.0%	100.0%
		T2	100.0%	100.0%	100.0%	100.0%
		T3	100.0%	100.0%	100.0%	100.0%
IgM	aCL	n	18	14	10	12
		T1	16.7%	40.0%	100.0%	25.0%
		T2	66.7%	75.0%	33.4%	100.0%
		T3	66.7%	60.0%	66.7%	75.0%
	aβ2-GPI	n	12	12	12	11
		T1	100.0%	100.0%	100.0%	75.0%
		T2	100.0%	100.0%	100.0%	100.0%
		T3	100.0%	100.0%	100.0%	100.0%

Detection rates when using the LIA for sera positive for criteria aPL in the comparative assays. n, number of positive sera in the respective comparative assay; T1, first tercile of the positive sera; T2, second tercile of the positive sera; T3, third tercile of the positive sera.

Considering non-criteria aPL (Table 2), the LIA correctly identified all sera positive for aPA and aPS IgG and IgM in the AES system regardless of their respective terciles. Positivity rates of the LIA for aPG, aPI, and aPT IgG as compared to AES increased from first to third tercile and were generally lower for aPT IgG (range: 22.2–55,6%) than for aPG and aPI (range: 80.0% - 100.0%). The same effect seems also to hold true for aPG, aPI, and aPT IgM. However, sound conclusions are impaired by the low number of positive sera here. Finally, none of the sera positive for aPC, aPE, and aAnnV IgG or IgM in the AES system exhibited a positive reaction in the respective field of the LIA.

**Table 2:** Detection of low, medium, and high positive sera for non-criteria aPL in the LIA.

aPL		AES IgG	AES IgM
aPA	n	17	8
	T1	100.0%	3 / 3
	T2	100.0%	2 / 2
	T3	100.0%	3 / 3
aPC	n	12	6
	T1	0.0%	0 / 2
	T2	0.0%	0 / 2
	T3	0.0%	0 / 2
aPE	n	19	9
	T1	0.0%	0 / 3
	T2	0.0%	0 / 3
	T3	0.0%	0 / 3
aPG	n	29	7
	T1	80.0%	3 / 3
	T2	100.0%	1 / 2
	T3	100.0%	1 / 2
aPI	n	22	9
	T1	87.5%	2 / 3
	T2	100.0%	2 / 3
	T3	100.0%	3 / 3
aPS	n	21	9
	T1	100.0%	3 / 3
	T2	100.0%	3 / 3
	T3	100.0%	3 / 3
aAnnV	n	18	8
	T1	0.0%	0 / 3
	T2	0.0%	0 / 2
	T3	0.0%	0 / 3
aPT	n	27	8
	T1	22.2%	0 / 3
	T2	55.6%	0 / 2
	T3	55.6%	1 / 3

Detection rates when using the LIA for sera positive for non-criteria aPL with the AES system. For non-criteria aPL with ≤ 10 positive sera in AES IgM the “number of positive sera in the LIA / number of positive sera in the respective tercile” is given for numeric reasons. n, number of positive sera in the AES assay; T1, first tercile of the positive sera; T2, second tercile of the positive sera; T3, third tercile of the positive sera.

### 3.3. Sensitivities and specificities for criteria aPL

Data on the diagnostic performances for criteria aPL are given in Table 3. Sensitivities of the LIA ranged from 0.67 for aCL IgM to 1.00 for aβ2-GPI IgG and IgM and were accompanied by specificities of 0.75 for aβ2-GPI IgG to 0.97 for aCL IgM. The ALE system exhibited a sensitivity of 0.88 for aβ2-GPI IgG and 1.00 for the other criteria aPL. Its specificities were 1.00 for aCL IgG and aβ2-GPI IgM and approximately 0.90 for aCL IgM and aβ2-GPI IgG. For the ACU system, sensitivities and specificities of approximately 0.85, 0.95, and 1.00 were observed for aCL IgG, aCL IgM, and aβ2-GPI IgM, respectively. Solely for aβ2-GPI IgG, the ACU’s sensitivity was somewhat higher than its specificity (1.00 vs. 0.85). With the UNI system, sensitivities and specificities for aβ2-GPI IgG and IgM of 1.00 were observed. Regarding aCL, the UNI yielded lower sensitivities of 0.65 and 0.83 than specificities of 0.92 and 1.00 for IgG and IgM, respectively. For the AES system a sensitivity of only 0.41 was calculated for aβ2-GPI IgG along with sensitivities of 0.83–1.00 for the other criteria aPL. Specificities of the AES system were high for aβ2-GPI IgG and IgM and aCL IgM (0.99–1.00) and slightly lower for aCL IgG (0.88).

**Table 3:** Criteria aPL: Sensitivities and specificities.

aPL		n	ALE [95% CI]	ACU [95% CI]	UNI [95% CI]	AES [95% CI]	LIA [95% CI]
Sensitivity							
IgG	aCL	53	1.00 [0.86–1.00]	0.84 [0.68–0.94]	0.65 [0.47–0.80]	0.97 [0.86–1.00]	0.92 [0.78–0.98]
	aβ2-GPI	53	0.88 [0.71–0.96]	1.00 [0.84–1.00]	1.00 [0.84–1.00]	0.41 [0.24–0.59]	1.00 [0.84–1.00]
IgM	aCL	53	1.00 [0.64–1.00]	0.92 [0.62–1.00]	0.83 [0.52–0.98]]	1.00 [0.64–1.00]	0.67 [0.35–0.90]
	aβ2-GPI	53	1.00 [0.64–1.00]	1.00 [0.64–1.00]	1.00 [0.64–1.00]	0.83 [0.52–0.98]	1.00 [0.64–1.00]
Specificity							
IgG	aCL	53	1.00 [0.90–1.00]	0.82 [0.69–0.91]	0.92 [0.81–0.98]	0.88 [0.76–0.95]	0.84 [0.71–0.93]
	aβ2-GPI	53	0.89 [0.78–0.96]	0.85 [0.73–0.94]	1.00 [0.90–1.00]	1.00 [0.90–1.00]	0.75 [0.61–0.85]
IgM	aCL	53	0.92 [0.83–0.97]	0.96 [0.89–0.99]	1.00 [0.93–1.00]	1.00 [0.93–1.00]	0.97 [0.91–1.00]
	aβ2-GPI	53	1.00 [0.93–1.00]	1.00 [0.93–1.00]	1.00 [0.93–1.00]	0.99 [0.93–1.00]	0.95 [0.87–0.99]

Sera were classified according to the manufacturers’ recommendations. Results of the LCA were considered as “gold standard”. CI, confidence interval.

### 3.4. Comparison of sensitivities and specificities for criteria aPL

Results of the comparison of sensitivities and specificities for the criteria aPL are depicted in Table 4 and in Supporting Information S2 and S3 Figs. With respect to the sensitive detection of aCL IgG, UNI performed significantly worse than any of the other investigated assays. Additionally, ACU exhibited a significantly lower sensitivity for aCL IgG than the most sensitive assay ALE. In contrast, the sensitivity of the AES for aβ2-GPI IgG was significantly lower than all other immunoassays. No significant differences were observed in the sensitivities for aCL and aβ2-GPI IgM. The specificity of ALE for aCL IgG was significantly higher than that of the ACU, AES, and LIA systems, but did not differ significantly from UNI. For aβ2-GPI IgG, the UNI and AES systems were equally specific. They were, however, significantly more specific than the three remaining assays (ALE, ACU, LIA). The most specific assays for aCL IgM (UNI, AES) were only significantly more specific than the ALE, but did not exhibit significant differences in between each other or in comparison to the ACU and LIA system. Specificities of aβ2-GPI IgM did not differ significantly.

**Table 4:** Criteria aPL: Comparison of sensitivities and specificities.

aPL		ALE	ACU	UNI	AES	LIA
aCL IgG	ALE		0.0412^a^	0.0009^a^	1.0000	0.2482
	ACU	0.0077^a^		0.0455^a^	0.0736	0.3711
	UNI	0.1336	0.1306		0.0015^a^	0.0094^a^
	AES	0.0412^a^	0.3711	0.6831		0.4795
	LIA	0.0133^a^	1.0000	0.1336	0.6171	
aCL IgM	ALE		1.0000	0.4795	con.	0.1336
	ACU	0.3711		1.0000	1.0000	0.3711
	UNI	0.0412^a^	0.2482		0.4795	0.6171
	AES	0.0412^a^	0.2482	con.		0.1336
	LIA	0.2207	1.0000	0.4795	0.4795	
aβ2-GPI IgG	ALE		0.1336	0.1336	0.0003^a^	0.1336
	ACU	0.7518		con.	< 0.0001^a^	con.
	UNI	0.0412^a^	0.0133^a^		< 0.0001^a^	con.
	AES	0.0412^a^	0.0133^a^	con.		< 0.0001^a^
	LIA	0.0801	0.1138	0.0005^a^	0.0005^a^	
aβ2-GPI IgM	ALE		con.	con.	0.4795	con.
	ACU	con.		con.	0.4795	con.
	UNI	con.	con.		0.4795	con.
	AES	1.0000	1.0000	1.0000		0.4795
	LIA	0.1336	0.1336	0.1336	0.3711	

Sensitivities without shading, specificities with gray shading. P-values calculated in the McNemar test.

^a^Significant p-values. Con., concordant.

Thus, the LIA performed significantly better in the sensitive detection of aCL IgG than the UNI and of aβ2-GPI IgG than the AES. Specificity for aCL IgG was significantly lower than the ALE and for aβ2-GPI IgG than the UNI and AES. No further significant differences in the sensitivities and specificities for criteria aPL between the LIA and ALE, ACU, UNI, and AES were observed.

### 3.5. Inter-rater agreement of LIA and AES for non-criteria aPL

With Cohen’s κ of 0.61–0.75, inter-rater agreement of LIA and AES for aPG and aPI IgG and IgM can be considered as good. For aPS, agreement was good for the IgM and moderate for the IgG isotype (Cohen’s κ 0.75 vs. 0.53, respectively). Similarly, a slightly lower Cohen’s κ of 0.34 indicating fair agreement was found for aPA IgG as compared to a Cohen’s κ of 0.43 for the IgM isotype suggesting moderate agreement. The reverse pattern was observed for aPT with Cohen’s κ 0.29 for IgG (fair agreement) vs. 0.10 for IgM (poor agreement). Cohen’s κ from -0.10 to 0.00 for aPC, aPE, and aAnnV IgG and IgM pointed to poor agreements. A very good agreement could not be demonstrated for any non-criterion aPL (Supporting Information S2 Table).

In consideration of the only modest agreement of both assay systems, application of β2-GPI as cofactor in the AES aPA, aPC, aPE, aPG, aPI, and aPS assays was further investigated. In the AES system, measured levels of these non-criteria aPL and the respective aβ2-GPI isotype exhibit a noteworthy positive correlation. With respect to the antibody isotype, the correlation seems to be more distinct for IgM than for IgG (Supporting Information S4 Fig).

### 3.6. Sensitivities and specificities for non-criteria aPL

Sensitivities for non-criteria aPL IgG for the LIA system ranged from 0.00 for aPC and aPE to 0.89 for aPA (Table 5). AES exhibited sensitivities in between 0.31 (for aPC IgG) and 0.58 (for aPG IgG) for non-criteria aPL. Sensitivities of the LIA for aPA, aPI, and aPS were significantly higher than AES (0.89 vs. 0.34, 0.66 vs. 0.44, and 0.81 vs. 0.42, respectively). Conversely, the AES ELISA turned out to be significantly more sensitive than the LIA for aPC (0.31 vs. 0.00), aPE (0.49 vs. 0.00), and aAnnV (0.45 vs. 0.08). Sensitivities for aPG and aPT IgG were not significantly different.

**Table 5:** Non-criteria aPL: Sensitivities, specificities, and comparison LIA vs. AES.

aPL		Sensitivity					Specificity				
		LIA		AES			LIA		AES		
		n	value [95% CI]	n	value [95% CI]	p	n	value [95% CI]	n	value [95% CI]	p
IgG	aPA	53	0.89 [0.77–0.96]	50	0.34 [0.21–0.49]	< 0.0001^a^	34	0.94 [0.80–0.99]	33	1.00 [0.85–1.00]	0.4795
	aPC	53	0.00 [0.00–0.10]	39	0.31 [0.17–0.48]	0.0015^a^	34	1.00 [0.85–1.00]	29	1.00 [0.83–1.00]	con.
	aPE	53	0.00 [0.00–0.10]	37	0.49 [0.32–0.66]	< 0.0001^a^	34	1.00 [0.85–1.00]	29	0.97 [0.82–1.00]	1.0000
	aPG	53	0.72 [0.58–0.83]	50	0.58 [0.43–0.72]	0.1138	34	1.00 [0.85–1.00]	33	1.00 [0.85–1.00]	con.
	aPI	53	0.66 [0.52–0.78]	50	0.44 [0.30–0.59]	0.0055^a^	34	0.94 [0.80–0.99]	33	1.00 [0.85–1.00]	0.4795
	aPS	53	0.81 [0.68–0.91]	50	0.42 [0.28–0.57]	< 0.0001^a^	34	1.00 [0.85–1.00]	33	1.00 [0.85–1.00]	con.
	aAnnV	53	0.08 [0.02–0.18]	40	0.45[0.29–0.62]	0.0056^a^	34	0.97 [0.85–1.00]	30	1.00 [0.83–1.00]	con.
	aPT	53	0.38 [0.25–0.52]	48	0.50 [0.35–0.65]	0.1456	34	0.88 [0.73–0.97]	32	0.91 [0.75–0.98]	1.0000
IgM	aPA	53	0.47 [0.33–0.61]	48	0.17 [0.07–0.30]	0.0005^a^	34	0.97 [0.85–1.00]	32	1.00 [0.84–1.00]	1.0000
	aPC	53	0.00 [0.00–0.10]	39	0.15 [0.06–0.31]	0.0412^a^	34	1.00 [0.85–1.00]	29	1.00 [0.83–1.00]	con.
	aPE	53	0.00 [0.00–0.10]	37	0.19 [0.08–0.35]	0.0233^a^	34	1.00 [0.85–1.00]	29	0.93 [0.77–0.99]	0.4795
	aPG	53	0.21 [0.11–0.34]	43	0.16 [0.07–0.31]	1.0000	34	1.00 [0.85–1.00]	29	1.00 [0.83–1.00]	con.
	aPI	53	0.21 [0.11–0.34]	48	0.19 [0.09–0.33]	1.0000	34	1.00 [0.85–1.00]	32	1.00 [0.84–1.00]	con.
	aPS	53	0.32 [0.20–0.46]	48	0.19 [0.09–0.33]	0.0736	34	1.00 [0.85–1.00]	32	1.00 [0.84–1.00]	con.
	aAnnV	53	0.08 [0.02–0.18]	44	0.18 [0.08–0.33]	0.2278	34	0.91 [0.76–0.98]	31	1.00 [0.84–1.00]	0.2482
	aPT	53	0.08 [0.02–0.18]	40	0.15 [0.06–0.30]	0.2888	34	0.94 [0.80–0.99]	30	0.93 [0.78–0.99]	1.0000

Sera were classified according to the manufacturers’ recommendations. Clinical diagnosis was considered as “gold standard”. P-values calculated in the McNemar test.

^a^Significant p-values. CI, confidence interval; con., concordant.

Amongst the non-criteria aPL IgM, sensitivities were again lowest for aPC and aPE (0.00) and highest for aPA (0.47) with the LIA. Sensitivities for the AES varied less and ranged from 0.15 for aPC and aPT IgM to 0.19 for aPE, aPI, and aPS IgM. The LIA was significantly more sensitive than AES for aPA IgM (0.47 vs. 0.17) and significantly less sensitive for aPC and aPE IgM (0.00 vs. 0.15 and 0.00 vs. 0.19, respectively). No further significant differences between the two investigated systems in the sensitivities for non-criteria aPL IgM were observed.

Specificities for the non-criteria aPL were ≥ 0.88 and ≥ 0.91 with the LIA and ≥ 0.91 and ≥ 0.93 with AES for the IgG and IgM isotype, respectively. Differences of specificities were not significant for any non-criteria aPL.

### 3.7. Clinical value of non-criteria aPL

Eleven (20.8%) out of the 53 APS-patients were positive for LA, but negative for aCL or aβ2-GPI IgG or IgM as concluded from LCA. When using the LIA, 9 (81.8%) of these patients exhibited positivity for any of the non-criteria aPL. All 9 patients were positive for aPA IgG. In addition, aPS IgG was found in 6, aPG IgG in 5, aPI IgG in 4, and aPT IgM in 2 samples and aAnnV IgG, aPT IgG, and aPA IgM in one sample each. With the AES assays, 6 (54.5%) of the 11 patients were positive for non-criteria aPL: in 4 samples solely aPT IgG or IgM was detected, in one sample aPT IgG and aPE IgM, and in one sample a combination of aPG IgG, aPI IgG, aPS IgG, and aAnnV IgG. All 6 samples positive for non-criteria aPL with the AES system were also found positive for non-criteria aPL in the LIA.

## 4. Discussion

The diagnostic performance of a novel LIA for the detection of aPL was investigated. Sensitivities and specificities of the LIA for criteria aPL are well comparable to established immunoassays. With respect to non-criteria aPL, the LIA offers advantages in the sensitive detection of aPL against anionic PLs whereas the classical ELISA exhibits strengths in the sensitive detection of aPL against neutral PLs.

Sensitivity of the LIA for aCL and aβ2-GPI IgG was significantly better than the worst and comparable to the three other established assays. Considering specificity for aCL and aβ2-GPI IgG, the LIA performed worse than the best and two best, but equal to three and two of the comparative assays, respectively. Moreover, with respect to aCL and aβ2-GPI IgM, no significant differences in sensitivity and specificity between the LIA and the established immunoassays were observed. As such, the diagnostic performance of the LIA for criteria aPL can be considered equal to the investigated established immunoassays.

In contrast, considerable differences in the results for non-criteria aPL obtained by LIA and an established ELISA system were demonstrated. This is illustrated by Cohen’s κ indicating very good agreement for none and good agreement for only 5, but poor agreement for 7 out of the 16 analytes. Differences are based on varying sensitivities for non-criteria aPL. Sensitivity of the LIA was significantly better for aPA, aPI and aPS IgG, and aPA IgM and approached significance for aPS IgM. The ELISA, however, demonstrated a significantly higher sensitivity for aPC and aPE IgG and IgM. Phosphatidic acid, phosphatidyl-serine, -glycerol, and -inositol carry a negative net charge at physiological pH. Phosphatidyl-choline and–ethanolamine in contrast constitute neutral molecules at pH 7.4. Thus, the LIA exhibits higher sensitivities for antibodies against anionic and the ELISA for antibodies against neutral PL. Since the ELISAs contained β2-GPI as cofactor apart from the respective PL as autoantigenic target, positive serum reactivity in these assays could be due to aβ2-GPI, too. With the AES system, aPA, aPC, aPE, aPG, aPI, and aPS indeed exhibited a positive correlation with aβ2-GPI in our cohort. However, it remains to be shown, whether the aPL reactivity in the respective ELISAs is based on β2-GPI binding alone. Thus, further studies are warranted to investigate whether this phenomenon can be explained by actual analytical interference or if the respective aPL arise concurrently for biological reasons.

The diagnostic performance of LIAs for aPL detection has been evaluated in two major studies: Egerer et al. [29] applied a shorter predecessor version and Roggenbuck et al. [27] the full-length LIA also investigated here. Our data not only confirm the comparable diagnostic performance of LIA and ELISA with respect to the criteria aPL, but also substantiate the findings of Egerer and Roggenbuck in several aspects. First, processed strips were read out manually (“eyeballing”) in the present study and not densitometrically with scanner and evaluation software as in the previous studies. The software, however, is not used in each routine laboratory. As such, our results are closer to the actual scenario in a diagnostic laboratory. Second, the aforementioned studies compared the determination of the criteria aPL via LIA only to a single ELISA system, which had been produced by the same manufacturer as the LIA. In our study, four comparative assays from a variety of manufacturers were included. And third, sensitivities and specificities for criteria aPL were calculated with respect to a LCA-derived gold standard. We introduced this approach recently [22] and consider it beneficial when evaluating agreement of aPL assays. A definitive diagnosis of APS requires positive laboratory criteria. Establishing the clinical diagnosis as gold standard is therefore inherently biased by the assays applied during the diagnostic work-up. The LCA-driven approach, however, does not require knowledge about the diagnosis: it merely evaluates whether the assay investigated agrees with the “consensus” opinion of already established assays regarding antibody-positivity or–negativity.

To the best of our knowledge, our work is the first study to compare non-criteria aPL detection of the LIA with established ELISAs thus revealing different strengths of the various assay surfaces. A LCA-based approached would clearly have been desirable here as well. A comprehensive investigation of all non-criteria aPL detected in the LIA, however, was only possible with one of the four assay systems that had been applied for the evaluation of the criteria aPL. We, therefore, choose to use the clinical diagnosis as the gold standard. One may argue that this approach is less critical for non-criteria aPL as they are not required for the diagnosis of APS.

Characteristics of patient and control group entail some limitations of our study. First of all, the limited sample size may impair generalizability of our data. Furthermore, a high percentage of double and triple positive subjects in our patient cohort may have increased the sensitivities for criteria aPL. And finally, the lack of disease controls may have yielded rather high specificities. As such, selection of patient and control groups may explain the mostly higher sensitivities and specificities in our study as compared to previous ones. From an analytical point of view, the time interval between the “old” ELISA measurements of the criteria aPL and the “new” measurements with the LIA and of non-criteria aPL via ELISA may introduce some bias into our work. If at all, this bias would be to the disadvantage of the LIA and should not result in a false-good evaluation of this assay.

Nevertheless, the observed high sensitivity of the LIA for aPL against anionic PLs sheds light on the optimal solid phases for testing of non-criteria aPL. The hydrophobic membrane of the LIA seems to be more suited for the detection of aPL against anionic PLs, whereas the polystyrene plastics of the ELISA plate detects neutral PLs with higher sensitivity. Of note, β2-GPI reactivity in these ELISAs cannot be excluded. Keeping in mind, that the most relevant autoantigenic epitopes for aPL are located on β2-GPI, our data support the hypothesis previously proposed [27, 30]: the porous hydrophobic polyvinylidene fluoride membrane of the LIA incorporates the hydrophobic tails of the PLs; only their hydrophilic and in part anionic moieties are exposed allowing optimal antigen presentation to aPL. The negative net charge of anionic PL favors binding of β2-GPI domain V resulting in optimal presentation of the immunodominant epitopes in domain I. In contrast, neutral PL do not bind domain V of β2-GPI in the LIA. Thus, the planar ELISA solid phase with immobilization of PLs along with β2-GPI as cofactor in random orientation may offer a more advantageous surface for aPL binding in this reaction environment. As such, tailoring the solid-phase to the respective PL rather than a “one size fits all” approach may be crucial to improve assay performance.

The LIA proved to be a suitable tool for detection of criteria and presumably also of non-criteria aPL. As compared to classical ELISAs, the LIA offers several interesting features: in one single run a comprehensive aPL profile can be generated considerably quicker and with less reagent costs and effort as compared to ELISAs as one LIA test strip replaces 10 single ELISAs; it is suited for low as well as for high sample throughput and requires only a basic laboratory equipment. Our data demonstrate that the majority of APS patients with LA but without criteria aPL exhibits positivity for non-criteria aPL–thereby pointing to possible diagnostic benefits of the determination of non-criteria aPL. Of note, strength of positivity for non-criteria aPL as well as the time course of titers were not considered in this study. As such, better availability and convenient analysis of aPL profiles may foster research in this area and help to better elucidate the clinical significance of non-criteria aPL.

In summary, our work demonstrates that the LIA exhibits a diagnostic performance comparable to established immunoassays for criteria aPL and offers advantages with respect to the detection of non-criteria aPL against anionic PL. It constitutes a valuable tool for aPL profiling in a clinical as well as in a research setting. Beyond that, our data will serve as a guide for assay developers when designing the solid-phase for detection of non-criteria aPL.

## Supporting information

S1 Fig Illustration of the statistical approach. For criteria aPL, the diagnostic performance was calculated against a “gold standard” derived from the results of the ALE, ACU, UNI, and AES systems via LCA. In contrast, the clinical diagnosis served as the gold standard to determine sensitivities and specificities for non-criteria aPL.
(PPTX)

S2 Fig Comparison of the sensitivities for criteria aPL. Sensitivities of the investigated assays for aCL IgG (A), aCL IgM (B), aβ2-GPI IgG (C), and aβ2-GPI IgM (D). Error bars denote a 95% confidence interval. Significant differences are marked with horizontal square brackets (*: 0.05 > p ≥ 0.01; **: 0.01 > p ≥ 0.001; ***: p < 0.001).
(TIF)

S3 Fig Comparison of the specificities for criteria aPL. Specificities of the investigated assays for aCL IgG (A), aCL IgM (B), aβ2-GPI IgG (C), and aβ2-GPI IgM (D). Error bars denote a 95% confidence interval. Significant differences are marked with horizontal square brackets (*: 0.05 > p ≥ 0.01; **: 0.01 > p ≥ 0.001; ***: p < 0.001).
(TIF)

S4 Fig Association of AES non-criteria aPL and AES aβ2-GPI. Levels of aPA, aPC, aPE, aPG, aPI, and aPS as a function of aβ2-GPI as measured with the AES system for IgG (A) and IgM (B) for all samples included in the study. AES aPE IgG: samples > 300 U/ml were not further diluted; AES aPG IgG and AES aPI IgG: samples > 315 U/ml were not further diluted.
(TIFF)

S1 Table Reference ranges of the comparative immunoassay systems. (DOCX)

S2 Table Non-criteria aPL: Inter-rater agreement LIA vs. AES. (DOCX)

S1 File Raw Data. Data underlying the findings described in the manuscript.
(CSV)

## Abbreviations

aAnnV: anti-annexin V
aβ2-GPI: anti-β2-glycoprotein I
aCL: anti-cardiolipin
ACU: ACL AcuStar
AES: AESKULISA
ALE: Alegria
aPA: anti-phosphatidic acid
aPC: anti-phosphatidyl-choline
aPE: anti-phosphatidyl-ethanolamine
aPG: anti-phosphatidyl-glycerol
aPI: anti-phosphatidyl-inositol
aPL: anti-phospholipid antibodies
aPS: anti-phosphatidyl-serine
APS: antiphospholipid syndrome
aPT: anti-prothrombin
CI: confidence interval
CLSI: Clinical and Laboratory Standards Institute
con: concordant
GA: Generic Assays
LA: lupus anticoagulant
LCA: latent class analysis
LIA: line immunoassay
PL: phospholipid
UNI: UniCAP 250
