# Supplementary material for: Evaluation of the sensitivity and specificity of a novel line immunoassay for the detection of criteria and non-criteria antiphospholipid antibodies in comparison to established ELISAs
Source: PLoS One. 2019 Jul 24;14(7):e0220033. doi: 10.1371/journal.pone.0220033 (PMC6655644; doi:10.1371/journal.pone.0220033)
Supplement: S1 Fig — For criteria aPL, the diagnostic performance was calculated against a “gold standard” derived from the results of the ALE, ACU, UNI, and AES systems via LCA. In contrast, the clinical diagnosis served as the gold standard to determine sensitivities and specificities for non-criteria aPL. (PPTX) [file pone.0220033.s001.pptx]

## Slide 1
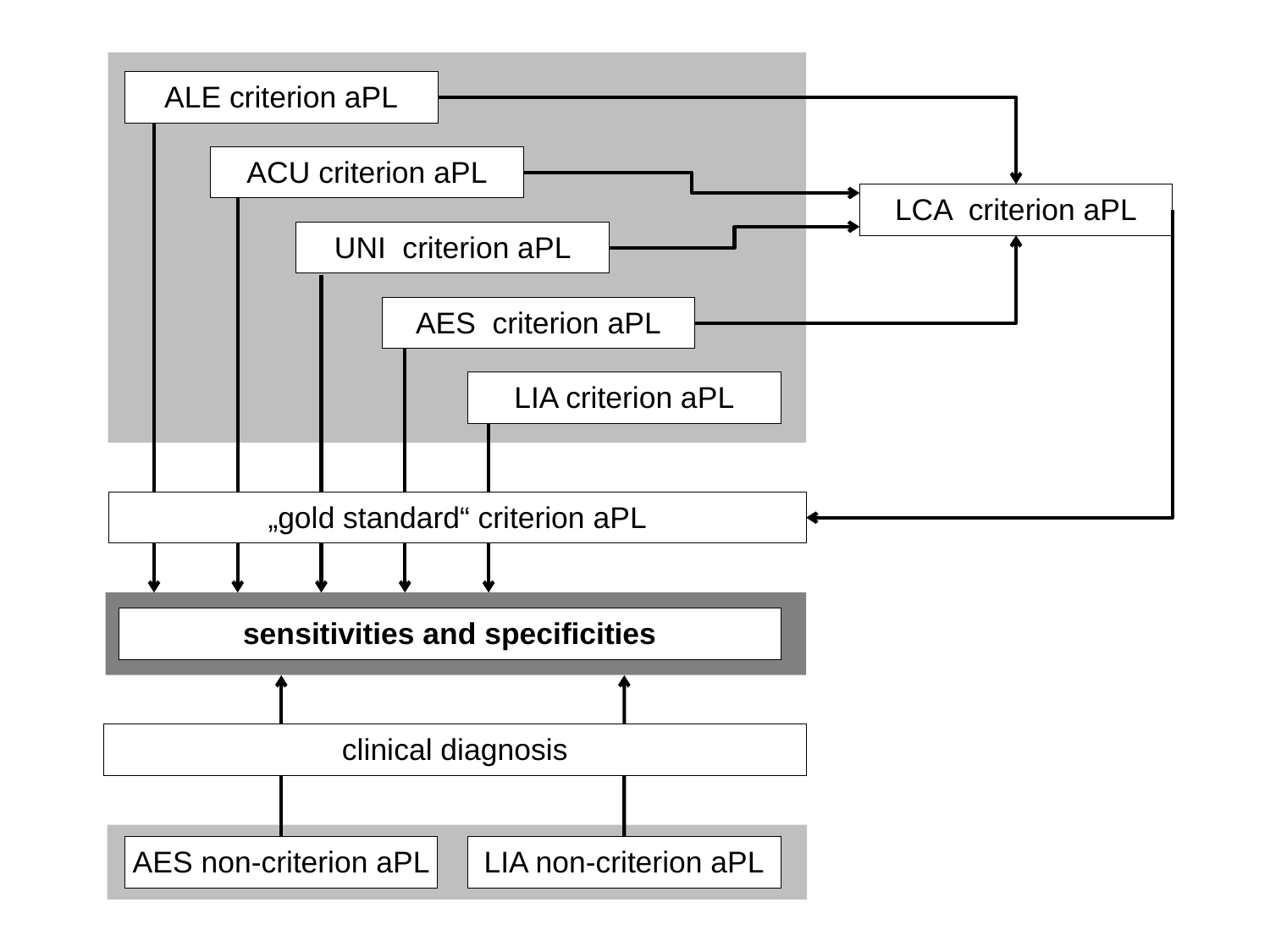

ALE criterion aPL
ACU criterion aPL
LCA criterion aPL
UNI criterion aPL
AES criterion aPL
LIA criterion aPL
„gold standard“ criterion aPL
sensitivities and specificities
clinical diagnosis
AES non-criterion aPL
LIA non-criterion aPL
