# Supplementary figures and images for: Evaluation of the sensitivity and specificity of a novel line immunoassay for the detection of criteria and non-criteria antiphospholipid antibodies in comparison to established ELISAs
Source: PLoS One. 2019 Jul 24;14(7):e0220033. doi: 10.1371/journal.pone.0220033 (PMC6655644; doi:10.1371/journal.pone.0220033)

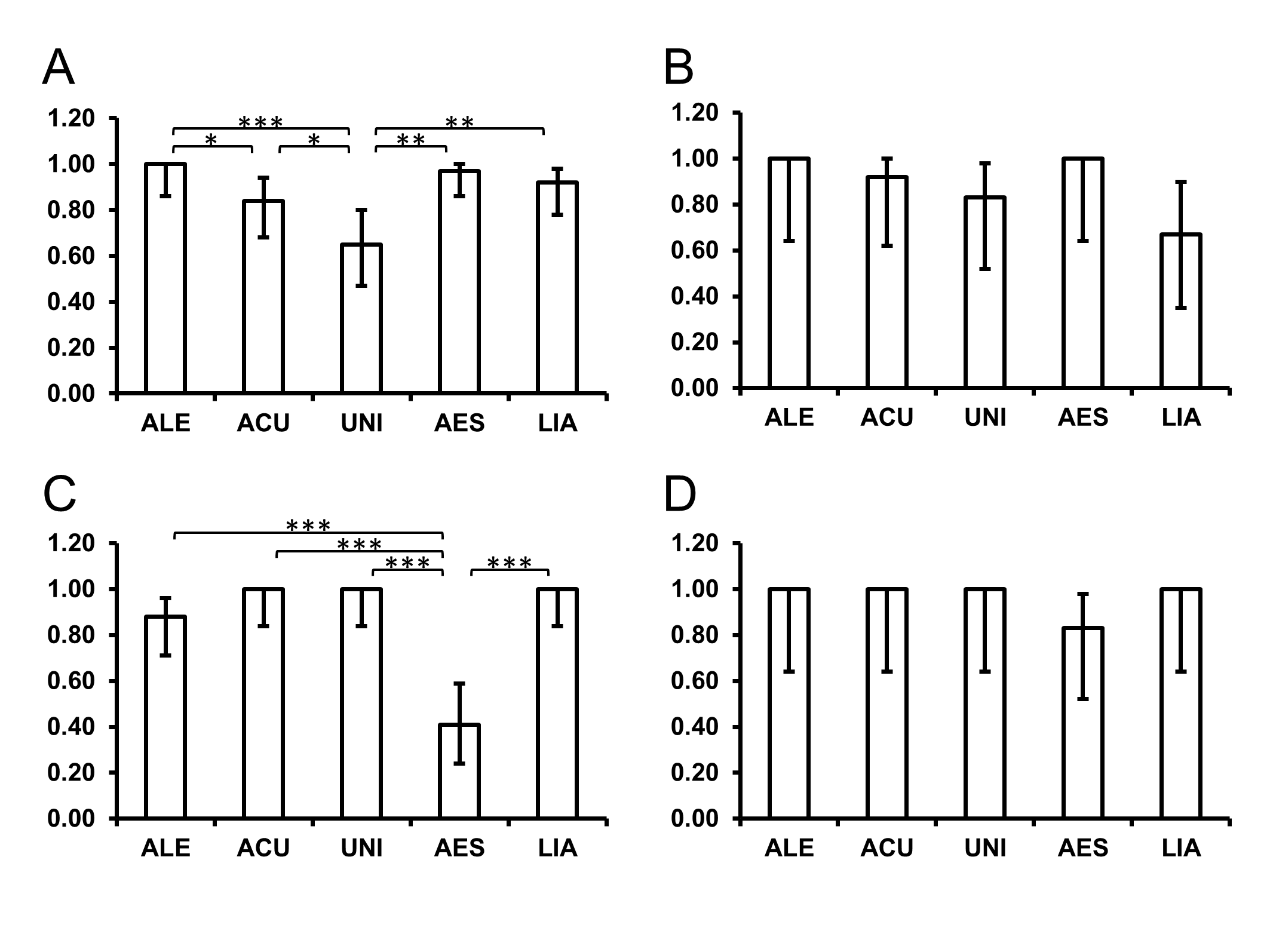

Supplement: S2 Fig — Sensitivities of the investigated assays for aCL IgG (A), aCL IgM (B), aβ2-GPI IgG (C), and aβ2-GPI IgM (D). Error bars denote a 95% confidence interval. Significant differences are marked with horizontal square brackets (*: 0.05 > p ≥ 0.01; **: 0.01 > p ≥ 0.001; ***: p < 0.001). (TIF) [file pone.0220033.s002.tif]

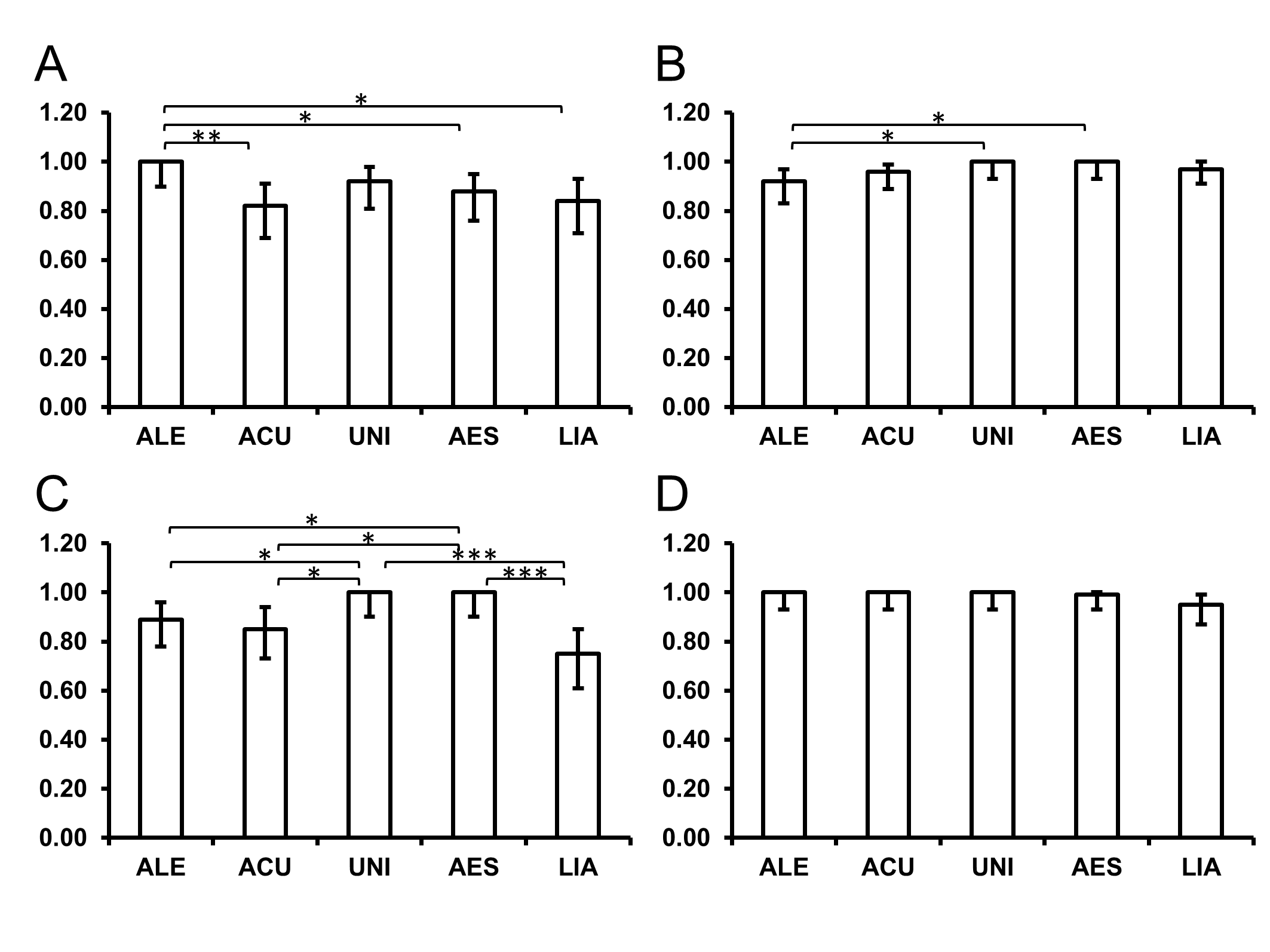

Supplement: S3 Fig — Specificities of the investigated assays for aCL IgG (A), aCL IgM (B), aβ2-GPI IgG (C), and aβ2-GPI IgM (D). Error bars denote a 95% confidence interval. Significant differences are marked with horizontal square brackets (*: 0.05 > p ≥ 0.01; **: 0.01 > p ≥ 0.001; ***: p < 0.001). (TIF) [file pone.0220033.s003.tif]
