# Supplementary material for: Evaluation of the sensitivity and specificity of a novel line immunoassay for the detection of criteria and non-criteria antiphospholipid antibodies in comparison to established ELISAs
Source: PLoS One. 2019 Jul 24;14(7):e0220033. doi: 10.1371/journal.pone.0220033 (PMC6655644; doi:10.1371/journal.pone.0220033)
Supplement: S1 Table — (DOCX) [file pone.0220033.s005.docx]

|  | ALE | ACU | UNI | AES |
| --- | --- | --- | --- | --- |
| aCL IgG | < 10 GPL-U/ml | ≤ 20 U/ml | < 10 GPL-U/ml  10 - 40 GPL-U/ml weak positive | < 12 GPL/ml  12 - 18 GPL/ml equivocal |
| aCL IgM | < 7 MPL-U/ml | ≤ 20 U/ml | < 10 MPL-U/ml  10 - 40 MPL-U/ml weak positive | < 12 MPL/ml  12 - 18 MPL/ml equivocal |
| aβ2-GPI IgG | < 5 U/ml  5 - 8 U/ml borderline | ≤ 20 U/ml | < 7 U/ml  7 - 10 U/ml equivocal | < 12 U/ml  12 - 18 U/ml equivocal |
| aβ2-GPI IgM | < 5 U/ml  5 - 8 U/ml borderline | ≤ 20 U/ml | < 7 U/ml  7 - 10 U/ml equivocal | < 12 U/ml  12 - 18 U/ml equivocal |
| non-criteria aPL IgG | - | - | - | < 12 U/ml  12 - 18 U/ml equivocal |
| non-criteria aPL IgM | - | - | - | < 12 U/ml  12 - 18 U/ml equivocal |
| calculation of cut-offs | 99 % percentile  healthy blood donors  (n = 300) | 99 % percentile  healthy blood donors  (n = 250 -262) | 99 % percentile healthy blood donors (n = 400) plus data of APS patients (n = 50 - 101)  plus data of disease controls (n = 351)  plus comparison with competitors’ assays | mean + 2 × standard deviation  healthy blood donors  (n ≥ 100) |
