# Supplementary material for: Evaluation of the sensitivity and specificity of a novel line immunoassay for the detection of criteria and non-criteria antiphospholipid antibodies in comparison to established ELISAs
Source: PLoS One. 2019 Jul 24;14(7):e0220033. doi: 10.1371/journal.pone.0220033 (PMC6655644; doi:10.1371/journal.pone.0220033)
Supplement: S2 Table — (DOCX) [file pone.0220033.s006.docx]

| aPL | Cohens κ | |
| --- | --- | --- |
|  | IgG | IgM |
| aPA | 0.34 | 0.43 |
| aPC | 0.00 | 0.00 |
| aPE | 0.00 | 0.00 |
| aPG | 0.75 | 0.63 |
| aPI | 0.61 | 0.75 |
| aPS | 0.53 | 0.75 |
| aAnnV | -0.10 | -0.10 |
| aPT | 0.29 | 0.10 |
